# Circulating miR-206, miR-181b, and miR-21 as promising biomarkers in hypothyroidism and their relationship to related hyperlipidemia and hepatic steatosis

**DOI:** 10.3389/fmolb.2024.1307512

**Published:** 2024-02-02

**Authors:** Asmaa Mohammed, Olfat G. Shaker, Mahmoud A. F. Khalil, Abeer K. Abu-El-Azayem, Amira Samy, Shaimaa A. Fathy, Mohamed M. K. AbdElguaad, Fatma A. M. Mahmoud, Randa Erfan

**Affiliations:** ^1^ Department of Medical Biochemistry and Molecular Biology, Faculty of Medicine, Fayoum University, Fayoum, Egypt; ^2^ Department of Medical Biochemistry and Molecular Biology, Faculty of Medicine, Cairo University, Cairo, Egypt; ^3^ Department of Microbiology and Immunology, Faculty of Pharmacy, Fayoum University, Fayoum, Egypt; ^4^ Department of Medical Microbiology and Immunology, Faculty of Medicine, Cairo University, Cairo, Egypt; ^5^ Department of Clinical and Chemical Pathology, Faculty of Medicine, Cairo University, Cairo, Egypt; ^6^ Department of Internal Medicine, Diabetes and Endocrinology, Faculty of Medicine, Cairo University, Cairo, Egypt; ^7^ Department of Physiology, Faculty of Medicine, Fayoum University, Fayoum, Egypt; ^8^ Department of Tropical Medicine, Faculty of Medicine, Fayoum University, Fayoum, Egypt

**Keywords:** thyroid hormone, hepatic steatosis, microRNA, miR-206, MiR-181b, miR-21

## Abstract

**Background:** Thyroid hormones (THs) signaling has profound effects on many physiological processes. The regulation of THs signaling in various tissues involves the action of microRNAs (miRNAs) on thyroid deiodinases and receptors. THs regulate the expression of certain miRNAs and their target messenger RNAs (mRNAs) in various tissues and cells. The modulation of miRNA levels by THs affects their functions in processes such as liver lipid metabolism, skin physiology, and muscle and heart performance.

**Aim:** This research aimed to investigate miR-181b, miR-206, and miR-21 in the serum of patients with subclinical and overt hypothyroidism to determine their possible role in the diagnosis of the disease and their relationship to clinical disorders related to hypothyroidism.

**Methods:** This study included ninety participants, divided evenly into three groups as follows: patients with overt hypothyroidism diagnosed clinically, radiologically, and by investigation, subclinical hypothyroid patients, and healthy volunteers. The patients had a thorough medical history and underwent a clinical examination. Laboratory tests included plasma cholesterol, LDL, HDL, TGs, liver and renal function tests, CBC, fasting insulin, HOMA-IR, HbA1c, TSH, and free T4. The serum levels of miR-21, miR-206, and miR-181b were measured using qRT-PCR.

**Results:** miR-206 and miR-181b levels were higher in the subclinical group, followed by the hypothyroid and control groups. For miR-21, there was a significantly lower mean value in both the hypothyroid and subclinical groups than in the control group, with no difference between the two groups. Both miR-206 and miR-181b showed a significant negative association with albumin and free T4 levels and a significant direct association with GGT, ALT, AST, creatinine, uric acid, TGs, TC, LDL, TSH, thyroid volume, and CAP score. The same correlation pattern was observed for miR-181b, except that it was not significantly correlated with the TGs. For miR-21 levels, there was a significant positive correlation with albumin, free T4 level, and kPa score and a negative correlation with GGT, ALT, AST, creatinine, uric acid, HOMA-IR, HbA1c, TC, LDL, TSH, and CAP score. Cases with F1 kPa score and S2 CAP scores had significantly higher averages for miR-206 and miR-181b, with a *p*-value of 0.05. Moreover, miR-21 levels were significantly lower in the S2 CAP score group.

**Conclusion:** These miRNAs (miR-206, miR-181b, and miR-21) may be used as diagnostic biomarkers for hypothyroidism. They may be used as therapeutic targets to control dyslipidemia and hepatic steatosis during hypothyroid disease.

## 1 Introduction

Primary hypothyroidism is a prevalent condition that depends on the dietary iodine intake, age, and sex of the population. The high sensitivity of the hypothalamic-pituitary axis to changes in blood thyroid hormone (TH) levels makes the serum thyroid-stimulating hormone (TSH) level a reliable indicator for diagnosing this condition (Chaker et al., 2017). Subclinical hypothyroidism is an early stage of thyroid failure characterized by elevated TSH and normal TH levels. This stage was initially defined as being asymptomatic. However, when TH levels drop below normal, overt hypothyroidism develops along with severe symptoms (Hegedus et al., 2022). Various clinical conditions such as abnormal blood lipid levels (van Vliet et al., 2021), non-alcoholic fatty liver (NAFLD) (Teimouri et al., 2022), increased insulin resistance (assessed by HOMA-IR) (Shehata et al., 2022), type 2 diabetes mellitus (T2DM) (Han et al., 2022), and impaired kidney function (Ammar et al., 2021) are also associated with thyroid dysfunction.

MicroRNAs (miRNAs) play crucial roles in gene regulation. These are short RNAs of approximately 23 nucleotides, derived from a precursor with a double-stranded or hairpin structure. miRNA biogenesis involves RNA polymerase II (Landthaler et al., 2004). miRNAs can inhibit gene expression and translation via RISC, which binds to the 3′-UTRs of target mRNAs (Bartel, 2009). miRNAs are associated with various metabolic disorders, such as obesity and diabetes (Ji and Guo, 2019). Hypothyroidism, for example, is influenced by several miRNAs, such as miR-29a-3p (Tokic et al., 2018). miR-224-5p may also affect the development of hypothyroidism by targeting deiodinases, which convert T4 to rT3 (Wang et al., 2020).

A previous study demonstrated that hepatic expression of miR-206, miR-133a, and miR-133b is modulated by TH status in mice (Visser et al., 2009). It was also shown that serum levels of miR-206 are reduced in hyperthyroid patients and that this miRNA participates in the T3-mediated regulation of lipid metabolism in HepG2 cells, a human liver cancer cell line. This study suggested a novel molecular mechanism for TH-mediated lipid metabolism (Zheng et al., 2018). MiR-181b is another miRNA overexpressed in papillary thyroid cancer (PTC) cells (Nikiforova et al., 2008). Li et al. demonstrated that miR-181b downregulation inhibited cell proliferation and induced apoptosis by increasing the expression of CYLD, a lysine 63 deubiquitinase.

Additionally, miR-181b expression was approximately eight-fold higher in tumor tissues than in normal tissues (Li et al., 2014). Moreover, miR-181b overexpression has been linked to an increased potential for cancer recurrence and lymph node metastasis (Pamedytyte et al., 2019). MiR-21 is a key miRNA involved in PTC pathogenesis. Its expression is altered in tumor tissues (Samsonov et al., 2016). Ortiz et al. revealed that hypomethylation of DNA leads to overexpression of miR-141b and miR-21, which results in reduced transcription of their target genes (Ortiz et al., 2018). Sondermann et al. revealed a positive association between low miR-21 expression and an increased PTC recurrence rate. The authors also identified miR-21 and miR-9 as strong predictors of PTC recurrence (Sondermann et al., 2015). In our study, we chose three miRNAs that are regulated by TH (miR-206, miR-181b, and miR-21) to examine their levels in patients with hypothyroidism and subclinical hypothyroidism. This is the first study to examine the fold changes of miR-206, miR-181b, and miR-21 in the serum of patients with subclinical and overt hypothyroidism to detect their possible role in the diagnosis of the disease and their relationship with the development of associated clinical disorders.

## 2 Methods

### 2.1 Ethical statement

This study followed the ethical principles established by the pertinent national and institutional committees on human experimentation in line with the revised 2008 Helsinki Declaration. Approval for all procedures involving human subjects or patients (code: R384) was obtained from the Research Ethics Committee of the General Organization for Teaching Hospitals and Institutes (GOTHI). All the patients provided written informed consent before participating in the study. The manuscript was prepared according to the Strengthening the Reporting of Observational Studies in Epidemiology (STROBE) guidelines (Moher et al., 2012).

### 2.2 Study design and patient selection

This research involved three groups. The first group included 30 patients with overt hypothyroidism diagnosed clinically, radiologically, or by investigation. The second group comprised 30 subclinical hypothyroid patients, and the last group consisted of 30 healthy volunteers.

The following definitions were used to define overt hypothyroidism and subclinical hypothyroidism patients:

The term “subclinical hypothyroidism” is used to define that grade of primary hypothyroidism in which there is an elevated TSH concentration in the presence of normal serum-free thyroxine (T4) and triiodothyronine (T3) concentrations. Subclinical hypothyroidism may progress to overt hypothyroidism in approximately 2%–5% of cases annually. At the same time, overt hypothyroidism is clear hypothyroidism characterized by an increased TSH and a decreased T4 level (Khandelwal and Tandon, 2012).

All patients were subjected to full history and clinical examination. Full history taking covered asking about symptoms suggesting hypothyroidism, including Fatigue, loss of energy, lethargy, weight gain, decreased appetite, cold intolerance, dry skin, hair loss, sleepiness, muscle pain, joint pain, weakness in the extremities, depression, emotional lability, mental impairment, forgetfulness, impaired memory, inability to concentrate, constipation, menstrual disturbances, impaired fertility, decreased perspiration, paresthesias, nerve entrapment syndromes, blurred vision, decreased hearing, fullness in the throat and hoarseness (Spanou et al., 2019).

Regarding physical examination, vital signs, including blood pressure, pulse, respiratory rate, and temperature, were assessed. A detailed general examination, head and neck, chest, heart, abdominal and neurological examination were performed to look for the following signs: weight gain, slowed speech and movements, dry skin (rarely, yellow-hued from carotene), Jaundice, pallor, coarse, brittle, straw-like hair, loss of scalp hair, axillary hair, pubic hair, or a combination, dull facial expression, coarse facial features, periorbital puffiness, macroglossia, goiter (simple or nodular), hoarseness, decreased systolic blood pressure and increased diastolic blood pressure, bradycardia, pericardial effusion, abdominal distention, ascites (uncommon), hypothermia (only in severe hypothyroid states), nonpitting edema (myxedema), pitting edema of lower extremities. Hyporeflexia with delayed relaxation, ataxia, or both (Piantanida et al., 2016).

### 2.3 Diagnosis of liver steatosis and fibrosis

Transient elastography (TE) using a FibroScan^®^ device (Echosens, Paris) was carried out to investigate liver stiffness (LS). TE was performed with a standard M probe, and an XL probe (for obese patients) (Myers et al., 2012). Investigations were performed through the intercostal spaces, where patients were lying in the dorsal decubitus position with the right arm in maximal abduction. Measurements were taken following an overnight fast. The tip of the probe is placed in contact with the intercostal skin through a coupling gel in the ninth to 11th intercostal space at the level where a liver biopsy would be conducted. After locating a section of the liver that is at least 6 cm thick and devoid of significant vascular structures, the operator initiates shots by pressing the probe button.

Measurement depth was between 25 and 65 mm below the skin surface. The success or failure of each measurement is determined by the software. The subsequent parameters are applied for validating successful measurements:

1) Number of shots ≥10.
2) Ratio of valid shots to the total number of shots ≥60%.
3) Interquartile range (IQR) less than 30% of the median liver stiffness measurement (LSM) value (IQR/LSM ≤30%).

The results are described in kilopascal (kPa). The median value of the successful measurements was representative of LS (Castéra et al., 2010)**.**

In NAFLD population:

F0, no fibrosis à <5.5 kPa.

F1, mild fibrosis à 5.5–8.0 kPa.

F2, moderate fibrosis à 8.0–10.0 kPa.

F3, severe fibrosis à 11–16 kPa.

F4, cirrhosis à >16 kPa (Kamali et al., 2019a).

CAP score:

A FibroScan^®^ device (Echosens, Paris) was performed to measure LS with the assessment of hepatic steatosis using CAP. TE was carried out with a standard M probe. CAP measurement depends on the attenuation of US propagation in liver tissue due to the presence of fat and is calculated via a sophisticated process. CAP is measured only on validated measurements of TE according to the same criteria used for LSM and on the same signals. This ensures that the operator obtains a liver ultrasonic attenuation simultaneously and in the same volume of liver parenchyma as the LSM. The cut-off points used to stage steatosis were S1 ≥ 222 dB/m, S2 ≥233 dB/m, and S3 ≥290 dB/m (Sasso et al., 2012)**.**

In NAFLD population:

S0, no steatosis à <237 dB/m.

S1, mild steatosis à 237–259 dB/m.

S2, moderate steatosis à 259–291 dB/m.

S3, severe steatosis à 291–400 dB/m (Kamali et al., 2019b).

### 2.4 Laboratory investigation

Liver functions included alanine aminotransferase (ALT), total bilirubin (diazo), albumin (BCP), gamma-glutamyl transferase (GGT) (enzymatic), and aspartate aminotransferase (AST), according to the methodology recommended by the International Federation of Clinical Chemistry (IFCC). Renal functions included Urea (enzymatic), Creatinine (Jaffé), and uric acid (uricase) on the Beckman Coulter AU analyzer (Beckman Coulter Inc., CA 92821, United States). The assessment of cholesterol levels followed the accuracy criteria of the National Cholesterol Education Program (NCEP), and enzymatic techniques were used on the same instrument to analyze low-density lipoprotein (LDL), triglycerides, and high-density lipoprotein (HDL).

Fasting insulin using a two-site sandwich immunochemiluminometric assay (ICMA) on ADVIA Centaur (Siemens Healthcare, 91052 Erlangen, Germany) and fasting blood glucose (hexokinase) were used to calculate the homeostatic model assessment for insulin resistance (HOMA-IR) formula. Hba1c (hemoglobin a1c) measurement was performed using high-performance liquid chromatography (Ion Exchange HPLC) on a Tosoh G8 Analyzer (TOSOH Bioscience, US), which is the gold standard method for Hba1c measurement.

A direct chemiluminescence TSH assay using third-generation acridinium ester technology (detection limit less than 0.01 mU/L) was used to measure TSH concentrations. Serum TSH and free T4 (fT4) levels were analyzed to assess thyroid function using a Siemens ADVIA Centaur. The TSH assay is a two-site sandwich method, and the FT4 test is a competitive immunoassay. A 10-mL blood sample was obtained from each patient using a vacutainer device. The blood samples were stored in tubes containing separator gels. The serum layer (top) and packed cells were separated using gels. After 15 min, the tubes were centrifuged at 4,000 *g* for 10 min. The serum was stored at −80 °C after separating it from the clotted blood and then used for RNA extraction (Bush et al., 2001).

### 2.5 RNA extraction

RNA extraction was conducted following the protocol provided by the miRNeasy extraction kit (Qiagen, Valencia, CA, USA) with a total sample volume of 100 µL of serum. Initially, the reaction mixture was treated with the QIAzol lysis reagent (500 µL) and allowed to stand for 5 min at RT. Subsequently, 100 µL chloroform was added, followed by vortexing for 15 s and incubation for 2–3 min at RT. Centrifugation was performed at 4 °C for 15 min at 120,00× *g*. Upon removing the top aqueous phase, 1.5 times the volume of 100% ethanol was added to the remaining mixture. Each 700 µL of this solution was then centrifuged for 15 s at 8,000× *g* and RT on an RNeasy Mini spin column and placed in a 2 mL collection tube. The mixture was eluted through each column, and 700 µL of buffer RW1 was added. Centrifugation was performed at RT for 15 s at a speed of 8,000× *g*. The column received 500 µL of buffer RPE and then underwent centrifugation at RT at 8,000× *g* for 15 s. To further purify the mixture, the same amount of buffer RPE was added, and the column was centrifuged for 2 min at RT at the same speed. Using a pipette, 50 µL of RNase-free water was applied directly to the column to elute RNA. Centrifugation was performed for 1 minute at 8,000× *g*. A DNase Max Kit (Qiagen, Valencia, CA, United States) was used to perform DNase treatment to remove any DNA before RNA was converted into cDNA by reverse transcription. A NanoDrop 2000 spectrophotometer (Thermo Scientific, Waltham, MA, United States) was used to measure the concentration and purity of the RNA at 260/280 nm.

### 2.6 Reverse transcription

A high-capacity cDNA reverse transcription kit (Applied Biosystems, Foster City, CA, USA) was used to convert 1 µg of RNA into cDNA following the manufacturer’s instructions. The reaction was incubated at 37 °C for 60 min, denatured at 95 °C for 5 min, and stored at 4 °C.

### 2.7 miRNA expression by real-time quantitative PCR

qRT-PCR was used to measure serum levels of miR181b-5p and miR21-5p. The miRCURY LNA SYBR Green PCR Kit (Qiagen, Valencia, CA, United States), miRCURY LNA miRNA PCR Assay (Qiagen, Valencia, CA, United States), and Rotor-Gene Q RT-PCR system (Qiagen, Germany) were used for analysis. Predesigned primers from Qiagen (Valencia, CA, United States) were used for target miRNAs and SNORD 68 (reference gene) (Ayeldeen et al., 2018), and primer assays for miR206 (catalog no. MS00003787), miR181b (catalog no. YP00204530), miR21 (Catalog no. YP00204230), and SNORD68 (catalog no. MS00033712). A modified thermal cycle was performed, with an initial denaturation step of 2 min at 95°C, followed by 40cycles. Each cycle consisted of 10 s denaturation at 95°C and 60 s of annealing/extension at 56°C. The 2^−ΔΔCT^ equation was used to calculate the serum fold-change of miR181b-5p and miR21-5p for each sample (Livak and Schmittgen, 2001).

Ct, ΔCt, ΔΔCt, and Fold Change (FC) have the following mathematical relationships:

ΔCt (patients) = Ct (miRNA)–Ct (housekeeping gene).

ΔCt (control) = Ct (miRNA)–Ct (housekeeping gene).

ΔΔCt (patients) = ΔCt (patients)–ΔCt (Control).

FC = 2^−ΔΔCT^miRNA overexpression was indicated when the calculated fold change was greater than one, whereas a fold change of less than one signified miRNA downregulation. In this context, because the -ΔΔCt value for control individuals is equal to zero and 2° equals one, the control value was assumed to be 1 (Santoro et al., 2016).

## 3 Results

There were no statistically significant differences (*p* > 0.05) in the demographic characteristics of the study groups (Table 1).

**TABLE 1:** Comparison of different study groups based on demographic characteristics.

Variables	Study groups						*p*-value
	Hypothyroidism (n = 30)		Sub-clinical (*n* = 30)		Control (*n* = 30)		
	Mean ± SD		Mean ± SD		Mean ± SD		
Age (years)	37.4 ± 9.9		33.6 ± 9.5		31.6 ± 11.3		0.09
BMI (kg/m^2^)	31.9 ± 5.4		31.6 ± 6.9		30.4 ± 6.5		0.6
Sex	No. (%)		No. (%)		No. (%)		
Female	26	86.7%	27	90%	26	86.7%	0.9
Male	4	13.3%	3	10%	4	13.3%	

BMI, body mass index; SD, standard deviation.

### 3.1 Laboratory investigations

The CBC investigations did not reveal any statistically significant differences (*p* > 0.05) among the study groups. Creatinine and uric acid levels were significantly higher in hypothyroidism and subclinical cases than in controls, with no differences between the two patient groups. Mean ALT and HOMA-IR levels were significantly higher in the hypothyroidism group than in the subclinical and control groups. GGT and HbA1c levels differed among the three study groups, with a higher mean in the hypothyroid group and a lower mean among the controls. AST showed a significant difference between the hypothyroid group and the controls, with a higher mean in the hypothyroid group. The hypothyroid and subclinical groups of cases had significantly higher mean TGs, TC, and LDL levels than the controls, with no difference between case subgroups. In addition, there was a significantly lower mean HDL level in the hypothyroid group than in the control group; however, there was no difference between the subclinical group and the controls. The hypothyroid and subclinical groups showed a significantly higher mean TSH level than the controls, with no difference between the case subgroups. In addition, there was a significant difference between all groups regarding the level of free T4, with a lower mean among the hypothyroids, followed by the subclinical group, compared with the controls. Regarding thyroid volume, there was a significant difference between all three groups, with a higher mean among the hypothyroids, followed by the subclinical group (Table 2).

**TABLE 2:** Laboratory investigations comparing the study groups.

Variables	Hypothyroidism (*n* = 30)	Sub-clinical (*n* = 30)	Control (*n* = 30)	*p*-value
	Mean ± SD	Mean ± SD	Mean ± SD	
Complete blood count				
HB	11.8 ± 1.5	12.1 ± 1.5	12.4 ± 1.3	0.2
MCV	79.5 ± 10.2	80.6 ± 6.04	83.7 ± 4.1	0.07
MCH	26.3 ± 3.3	26.6 ± 2.7	27.9 ± 1.7	0.06
PLT	278.6 ± 90.6	299.2 ± 82.6	296.7 ± 61.8	0.6
Liver function tests				
T. billirubin	0.59 ± 0.26	0.57 ± 0.17	0.49 ± 0.09	0.1
Albumin	4.2 ± 0.27	4.3 ± 0.26	4.6 ± 0.27	0.6^b^
				<0.00^a^ ^,^ ^c^ ^,^ ^d^
GGT	34.5 ± 18.3	22.6 ± 8.8	13.2 ± 3.9	<0.001^a^ ^,^ ^b^ ^,^ ^c^
				0.01^a^ ^,^ ^d^
ALT	26.6 ± 19.4	18.8 ± 7.5	12.8 ± 4.3	0.04^a^ ^,^ ^b^
				<0.001^a^ ^,^ ^c^
				0.2^d^
AST	28.1 ± 19.02	20.9 ± 7.2	15.3 ± 5.5	0.07^b^
				<0.001^a^ ^,^ ^c^
				0.2^d^
Kidney function tests				
Urea	23.5 ± 9.8	23.4 ± 6.8	19.9 ± 4.6	0.1
Creatinine	0.82 ± 0.19	0.77 ± 0.11	0.65 ± 0.12	0.7^b^
				<0.001^a^ ^,^ ^c^
				0.005^a^ ^,^ ^d^
Uric acid	4.8 ± 1.3	4.4 ± 1.2	2.9 ± 0.38	0.3^b^
				<0.001^a^ ^,^ ^c^ ^,^ ^d^
Insulin profile				
Fasting insulin	13.1 ± 8.7	11.7 ± 9.4	8.6 ± 7.8	0.1
HOMA-IR	2.9 ± 2.1	1.9 ± 1.3	1.2 ± 0.58	0.04^a^ ^,^ ^b^
				<0.001^a^ ^,^ ^c^
				0.2^d^
HbA1c	5.4 ± 0.36	5 ± 0.33	4.7 ± 0.26	<0.001^a^ ^,^ ^b^ ^,^ ^c^ ^,^ ^d^
Lipid profile				
TGs	132.7 ± 56.1	103.4 ± 48.5	73.4 ± 33.8	0.06^b^
				<0.001^a^ ^,^ ^c^
				0.04^a^ ^,^ ^d^
TC	203.9 ± 50.7	188.8 ± 40.1	158 ± 28.7	0.5^b^
				<0.001^a^ ^,^ ^c^
				0.01^a^ ^,^ ^d^
LDL	135.5 ± 48.1	119.3 ± 37.04	91.6 ± 28.8	0.3^b^
				<0.001^a^ ^,^ ^c^
				0.02^a^ ^,^ ^d^
HDL	42.1 ± 8.2	49.1 ± 11.3	53.9 ± 13.5	0.06^b^
				<0.001^a^ ^,^ ^c^
				0.3^d^
Thyroid hormone profile				
TSH	64.9 ± 87.8	8.2 ± 2.8	1.7 ± 0.75	<0.001^a^ ^,^ ^b^ ^,^ ^c^
				0.9^d^
Free T4	0.56 ± 0.16	1.07 ± 0.13	1.26 ± 0.13	<0.001^a^ ^,^ ^b^ ^,^ ^c^ ^,^ ^d^
Thyroid volume	6.9 ± 3.3	5.3 ± 2.5	2.8 ± 1.1	0.04^a^ ^,^ ^b^
				0.001^a^ ^,^ ^c^ ^,^ ^d^

HB: hemoglobin; MCV: mean corpuscular volume; MCH: mean corpuscular hemoglobin; PLT: platelet; GGT: gamma-glutamyl transferase; ALT: alanine amino transferase; AST: aspartate amino transferase; HOMA-IR: homeostatic model assessment for insulin resistance; HbA1c: hemoglobin A1c; TGs: triglycerides; TC: total cholesterol; LDL: low-density lipoprotein; HDL: high-density lipoprotein. TSH: thyroid-stimulating hormone; free T4: free thyroxine 4.

^a^ Significant with ANOVA, test.

^b^ Comparison between hypothyroidism and subclinical groups.

^c^ Comparison between hypothyroidism and control groups.

^d^ Comparison between subclinical and control groups.

### 3.2 Radiological findings

The hypothyroid and subclinical groups of cases showed a significantly higher mean CAP score and a higher percentage of F1 levels by FibroScan than controls, with no difference between case subgroups. In addition, there was a significant difference between all three groups, with a higher percentage of S3 CAP scores among patients with hypothyroidism and S2 and S3 CAP scores among the subclinical group compared to controls who showed the S0 CAP scores (Table 3).

**TABLE 3:** Comparison of study groups based on radiological findings.

Variables	Study groups						*p*-value
	Hypothyroidism (*n* = 30)		Sub-clinical (*n* = 30)		Control (*n* = 30)		
	Mean ± SD		Mean ± SD		Mean ± SD		
CAP score	261.4 ± 77.9		247.9 ± 75.2		183.5 ± 36.4		0.9^c^
							0.001^a^ ^,^ ^d^ ^,^ ^e^
kPa score	4.1 ± 1.4		4.4 ± 1.4		4.6 ± 0.95		0.2
FibroScan	No. (%)		No. (%)		No. (%)		
F0	24	80%	23	76.7%	30	100%	0.7^c^
F1	6	20%	7	23.3%	0	0%	0.02^b^ ^,^ ^d^ ^,^ ^e^
CAP score							
S0	8	26.7%	13	43.3%	30	100%	0.03^b^ ^,^ ^c^
S1	6	20%	1	3.3%	0	0%	
S2	2	6.7%	7	23.3%	0	0%	<0.001^b^ ^,^ ^d^ ^,^ ^e^
S3	14	46.7%	9	30%	0	0%	

CAP, score: Controlled Attenuation Parameter; kPa score: kilopascals.

^a^ Significant with ANOVA.

^b^ Significant with chi-squared test.

^c^ Comparison between hypothyroidism and subclinical groups.

^d^ Comparison between hypothyroidism and control groups.

^e^ Comparison between subclinical and control groups.

### 3.3 Serum levels of biomarkers

**FIGURE 1:**
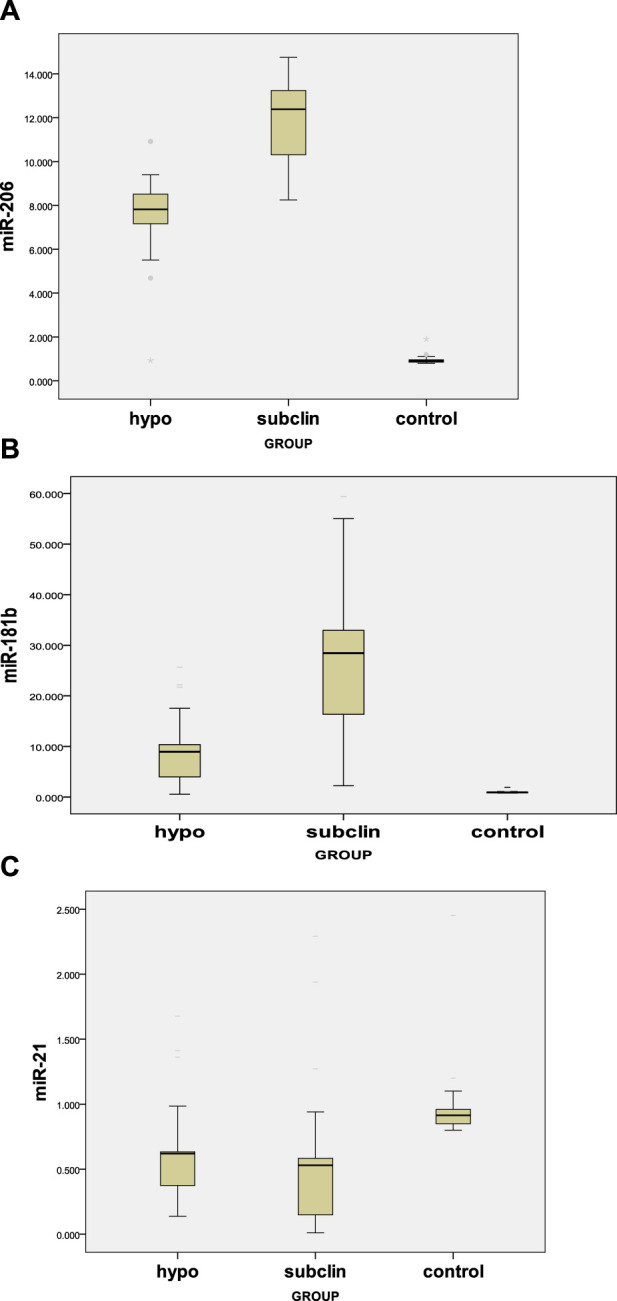
**(A)** Serum miR-206 levels in hypothyroid, subclinical, and control groups. **(B)** Serum miR-181b levels in hypothyroid, subclinical, and control groups. **(C)** Serum levels of miR-21 in the hypothyroid, subclinical, and control groups.

Our results demonstrated a significant difference (*p* < 0.05) between the three study groups concerning miR-206 and miR-181b, with a higher level among the subclinical group, followed by the hypothyroid group, and then the control group. For miR-21, there was a significantly lower mean value among both the hypothyroid and subclinical groups than in the control group, with no difference between the two groups (Table 4; Figures 1A–C).

**TABLE 4:** Comparison of markers.

Variables	Hypothyroidism (*n* = 30)	Sub-clinical (*n* = 30)	Control (*n* = 30)	*p*-value
	Mean ± SD	Mean ± SD	Mean ± SD	
miR-206	7.5 ± 1.8	11.9 ± 1.8	0.94 ± 0.20	<0.001^a^ ^,^ ^b^ ^,^ ^c^
miR-181b	9.2 ± 6.6	28.5 ± 14.4	0.94 ± 0.20	<0.001^a^ ^,^ ^b^
				0.002^c^
miR-21	0.62 ± 0.36	0.54 ± 0.52	0.94 ± 0.20	0.9^a^
				0.005^b^
				<0.001^c^

^a^ Comparison between hypothyroidism and subclinical groups.

^b^ Comparison between hypothyroidism and control groups.

^c^ Comparison between subclinical and control groups.

### 3.4 Comparison of biomarker levels in different demographic and radiological data among the patient group

For cases with an F1 kPa score and S2 CAP score, the patient group had a substantially greater mean value of miR-206 and miR-181b (*p* = 0.05). Additionally, miR-21 levels were significantly lower in the S2 CAP scores. However, there were no sex differences for any of these markers (Table 5).

**TABLE 5:** Comparison of markers in different demographic and radiological data among the patient group.

Variables	miR-206		*p*-value	miR-181b		*p*-value	miR-21		*p*-value
	Median	IQR		Median	IQR		Median	IQR	
Sex									
Female	7.9	9.9	0.6	9.1	23.7	0.6	0.62	0.45	0.6
Male	6.4	11.5		5.03	11.6		0.84	0.69	
kPa score									
F0	7.3	8.6	0.002*	5.9	18.7	0.004*	0.63	0.49	0.7
F1	10.9	4.7		16	21.6		0.62	0.53	
CAP score									
S0	0.98	8.5	0.001**	0.99	11.7	0.001**	0.86	0.37	0.02**
S1	8	2.2		3.9	8.4		0.62	0.24	
S2	12.7	4.6		28.5	42.1		0.58	0.43	
S3	8.5	3.5		15.7	18.5		0.62	0.42	

CAP, score: Controlled Attenuation Parameter; kPa score: kilopascals *: Significant with Mann–Whitney test; **: Significant with the KrusKal–Wallis test.

### 3.5 Correlation between biomarker levels and study variables

The patient group demonstrated a significant negative association between albumin, miR-206, and free T4 levels and a significant positive association between miR-206 and several parameters, such as GGT, ALT, AST, creatinine, uric acid, HOMA-IR, HbA1c, TGs, TC, LDL, TSH, thyroid volume, and CAP score. Similarly, miR-181b showed a significantly negative association with albumin and free T4 levels and a significantly positive association with various parameters, such as GGT, ALT, AST, creatinine, uric acid, HbA1c, TGs, TC, LDL, TSH, thyroid volume, and cap score. For miR-21 level, there was a significant positive correlation with albumin, free T4 level, and kPa score and a negative correlation with GGT, ALT, AST, creatinine, uric acid, HOMA-IR, HbA1c, TC, LDL, TSH, and CAP score.

The expression levels of miR-206 and miR-181b showed a strong positive association, while miR-21 exhibited a strong negative association with miR-181b and miR-21, as indicated by the correlation coefficients in Table 6.

**TABLE 6:** Relationship between study variables and biomarker levels in the patient group.

Variables	Study group					
	miR-206		miR-181b		miR-21	
	R	*p*-value	r	*p*-value	r	*p*-value
Age (years)	0.06	0.6	0.14	0.2	−0.05	0.6
BMI	0.12	0.3	0.10	0.3	−0.09	0.4
HB	0.02	0.9	0.04	0.7	−0.05	0.6
MCV	−0.12	0.2	−0.07	0.5	0.09	0.4
MCH	−0.09	0.4	−0.06	0.6	0.04	0.7
PLT	−0.08	0.5	−0.04	0.7	0.12	0.3
T. bilirubin	0.17	0.1	0.14	0.2	−0.14	0.2
Albumin	−0.37	0.001*	−0.36	0.001*	0.32	0.002*
GGT	0.37	0.001*	0.30	0.005*	−0.28	0.01*
ALT	0.32	0.002*	0.32	0.002*	−0.38	0.001*
AST	0.32	0.003*	0.31	0.003*	−0.52	0.001*
Urea	0.15	0.2	0.16	0.1	−0.20	0.05
Creatinine	0.32	0.002*	0.29	0.005*	−0.38	0.001*
Uric acid	0.45	0.001*	0.41	0.001*	−0.31	0.003*
Fasting insulin	0.20	0.06	0.12	0.3	−0.10	0.3
HOMA-IR	0.24	0.02*	0.17	0.1	−0.15	0.1
HbA1c	0.34	0.001*	0.33	0.001	−0.36	0.001*
TGs	0.24	0.02*	0.24	0.03*	−0.14	0.2
TC	0.31	0.003*	0.35	0.001*	−0.22	0.03*
LDL	0.29	0.004*	0.35	0.001*	−0.24	0.02*
HDL	−0.18	0.09	−0.16	0.1	0.12	0.3
TSH	0.42	0.001*	0.42	0.001*	−0.44	0.001*
Free T4	−0.29	0.005*	−0.33	0.001*	0.33	0.001*
Thyroid volume	0.34	0.001*	0.39	0.001*	−0.18	0.08
CAP score	0.35	0.001*	0.32	0.002*	−0.24	0.02*
kPa score	−0.05	0.7	−0.002	0.9	0.28	0.008*
miR-181b	0.91	0.001*	----	----	----	----
miR-21	−0.48	0.001*	−0.46	0.001*	----	----

BMI: body mass index; HB: hemoglobin; MCV: mean corpuscular volume; MCH: mean corpuscular hemoglobin; PLT: platelet; GGT: gamma-glutamyl transferase; ALT: alanine amino transferase; AST: aspartate amino transferase; HOMA-IR: homeostatic model assessment for insulin resistance; HbA1c: hemoglobin A1c; TGs: triglycerides; TC: total cholesterol; LDL: low-density lipoprotein; HDL: high-density lipoprotein. TSH: thyroid-stimulating hormone; free T4: free thyroxine4; CAP, score: Controlled Attenuation Parameter; kPa score: kilopascals *Significant with Spearman’s rho correlation test.

### 3.6 Detection of predictive power of miR-206, miR-181b, and miR-21 in hypothyroidism and subclinical cases

The sensitivity and specificity test for the diagnosis of hypothyroidism showed higher sensitivity and specificity among the miR-206, miR-181b, and miR-21 marker levels, with a sensitivity of 96.7%, 93.3%, and 83.3%, respectively, and a specificity of 96.7%, 96.7%, and 90% at a cut-off value of 1.55, 1.45, and 0.8 respectively.

**FIGURE 2:**
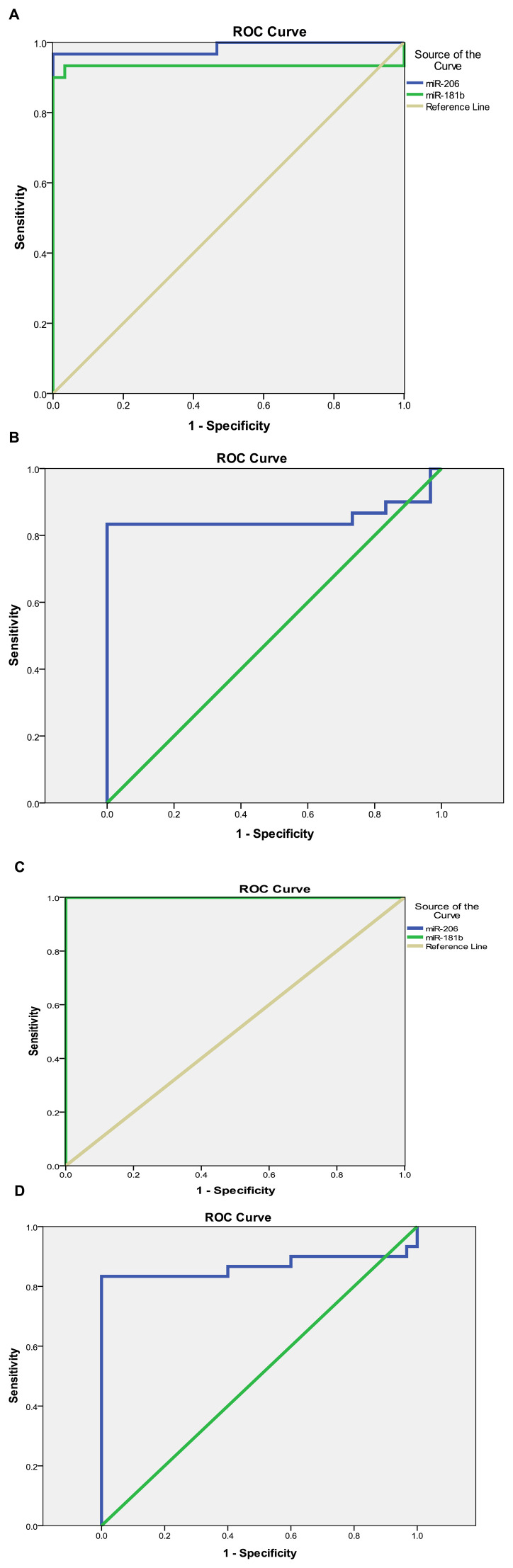
(Continued).

For subclinical cases, the sensitivity test for miR-206, miR-181b, and miR-21 showed a sensitivity of 100% and 83.3% and specificity of 96.7% and 100% at a cut-off value of 1.55 and 0.77, respectively (Table 7; Figures 2A–D).

**TABLE 7:** Diagnostic accuracy of marker levels for hypothyroidism and its subclinical cases measured by sensitivity and specificity.

Variable	Sensitivity (%)	Specificity (%)	AUC (%)	Cut-off point	*p*-value (CI)
Hypothyroidism group					
miR-206	96.7	96.7	98.4	1.55	<0.001 (0–100)
miR-181b	93.3	96.7	93.2	1.45	<0.001 (0–100)
miR-21	83.3	90	85.1	0.805	<0.001 (73–97.2)
Subclinical group					
miR-206	100	96.7	100	1.55	<0.001 (0–100)
miR-181b	100	96.7	100	1.55	<0.001 (0–100)
miR-21	83.3	100	86.8	0.773	<0.001 (75.5–98)

## 4 Discussion

THs modulate the expression levels of certain miRNAs and their target mRNAs in different cell types and organs. Many miRNAs contain thyroid hormone elements (TREs) in their regulatory regions, which are transcriptionally regulated by hormone-bound receptors. THs exert significant effects on processes, including lipid and liver metabolism, muscle and heart function, and skin physiology (Aranda, 2021), by altering the levels of miRNAs. Among these miRNAs, we selected miR-206, miR-181b, and miR-21, which have been reported to affect lipid metabolism in multiple disorders (Wang et al., 2017; Zheng et al., 2018; Zhao et al., 2021).

Our findings demonstrated that miRNAs in patients with thyroid illness were dysregulated compared to those in healthy controls. Regarding miR-206, a significant difference was found between the study groups (*p* < 0.05), with the subclinical group having the highest levels, followed by the hypothyroid and control groups. miR-206 was found to have a significantly higher mean among cases with F1 kPa score and S2 CAP score. Moreover, a significant inverse association between miR-206 and albumin and free T4 levels was observed in the patient group, as was a positive correlation between miR-206 and several biochemical markers (GGT, ALT, AST, creatinine, uric acid, HOMA-IR, HbA1c, TGs, TC, LDL, TSH, thyroid volume, and CAP score). Recent research has reported the contribution of thyroid hormones to the regulation of miR-206 in the mouse liver and corresponding cell line models (Dong et al., 2010) and in human skeletal muscle in hypothyroid patients (Visser et al., 2009). Thyroid hormones and their analogs reduce miR-206 levels, whereas hypothyroidism enhances its expression (Visser et al., 2009; Dong et al., 2010). Therefore, miR-206 is also suggested to act as a key mediator of thyroid hormone function since some of its targets (e.g., Mup1 and Gpd2) were shown to be modulated by thyroid hormones (Zhao et al., 2021). Zheng and colleagues showed the involvement of miR-206 in lipid metabolism in hyperthyroidism and hypothyroidism by finding that serum miR-206 expression decreases in hyperthyroid patients. They suggested that miR-206 was involved in regulating lipid metabolism by T3 in HepG2 cells, implying that miR-206 may play a role in thyroid hormone-related liver lipid metabolism disorders.

Additionally, miR-206 overexpression in HepG2 cells increased lipid accumulation and attenuated the T3-mediated decrease in TGs, and hepatic steatosis was associated with TH deficiency (Zheng et al., 2018). This previous study is consistent with ours, as we observed a positive correlation between miR-206 and GGT, ALT, AST, TGs, TC, and LDL levels. In addition, our findings confirmed the role of this miRNA in hepatic steatosis during hypothyroidism, as we found a positive correlation between miR-206 and the CAP score, which measures the percentage of fatty change in the liver. Additionally, we found a significantly higher mean miR-206 level in patients with an F1 kPa score than in those with an F0 kPa score, which measures the degree of liver stiffness.

Our findings revealed an inverse association between miR-206 and free T4 levels and a positive association between miR-206 and TSH levels. These results support those of previous studies, suggesting the influence of thyroid hormones on regulating miR-206 expression. In agreement with Mugdha et al., our results align with their finding that there is no relationship between the transcripts miR-206 and glucokinase in human islets, contrasting with the situation observed in mouse islets (Joglekar et al., 2018). The results reported by Vinod et al., who suggested that miR-206 regulated glucokinase mRNA post-transcriptionally in mouse pancreatic islets, indicating a possible role of miR-206 in glucose homeostasis regulation, possibly through glucokinase (Vinod et al., 2016), are inconsistent with our findings. In our study, a positive association was found between miR-206 and HbA1c as well as HOMA-IR, which further supports the potential involvement of miR-206 in metabolic processes.

Regarding miR-181b, we found that subclinical hypothyroid patients had the highest levels, followed by overt hypothyroid patients. Similar to our findings, Huang et al. discovered that thyroid hormones downregulate several miRNAs, including miR-200, miR-92a, and miR-181b (Huang et al., 2019).

The role of miR-181b in NAFLD and its direct interaction with NAD-dependent deacetylase sirtuin1 (SIRT1) were examined by Wang et al. They found that human patients with NAFLD and NAFLD mouse models induced by high-fat diet (HFD) had higher miR-181b levels and lower SIRT1 levels. They also showed that miR-181b directly targeted SIRT1 in HepG2 cells and that miR-181b inhibition reduced hepatic steatosis both *in vitro* and *in vivo*. Furthermore, SIRT1 overexpression reversed the effects of miR-181b on steatosis. Their data suggested that miR-181b may be involved in lipid metabolism dysregulation in NAFLD (Wang et al., 2017). Moreover, they discovered that the SIRT1, a direct target of miR-181b, modulated TGs production in human liver cells and influenced lipid metabolism. This previous study may explain our results, as we demonstrated a significant positive correlation between miR-181b and GGT, ALT, AST, HbA1c, TGs, TC, LDL, and CAP scores. In addition, miR-181b had a significantly higher mean in cases with F1 kPa score and S2 CAP score.

For miR-21, there was a significantly lower mean value in both the hypothyroid and subclinical groups than in the control group, with no difference between the two groups. For miR-21 levels, there was a significant positive correlation with albumin, free T4 level, and kPa score and a negative correlation with GGT, ALT, AST, creatinine, uric acid, HOMA-IR, HbA1c, TC, LDL, TSH, and CAP score. The present study agrees with the results of Huang et al., who reported that miR-21 expression is regulated by TH *in vivo* and *in vitro*. They showed that miR-21 levels were lower in the livers of rats with hypothyroidism and higher in rats with hyperthyroidism. They also demonstrated that tumor growth and metastasis of a TRα-overexpressing cell line were influenced by the thyroid status of the mice, with hyperthyroid mice having higher miR-21 and lower TIAM Rac1-associated GEF 1 (TIAM1) expression than euthyroid or hypothyroid mice. This confirmed that TH modulates miR-21 expression and its downstream target, TIAM1 (Huang et al., 2013).

Moreover, miR-21 has been widely investigated in thyroid cancers and has been identified as one of the miRNAs that could improve the diagnostic accuracy of PTC and is associated with TNM stage, metastasis, and tumor size. It can be used as a diagnostic marker to distinguish PTC from benign lesions (Wang et al., 2019; Samsonov et al., 2016; Zhang et al., 2017). MiR-21 was negatively correlated with GGT, ALT, AST, TC, LDL, and CAP scores. This finding aligns with prior research indicating downregulation of miR-21 expression in individuals affected by non-alcoholic fatty liver disease or in mice subjected to a HFD. Additionally, ablation of miR-21 has been observed to induce hepatic steatosis, expedited atherosclerosis, plaque necrosis, and vascular inflammation. These observations are consistent with and reinforce the conclusions of previous studies (Sun et al., 2015). Zhou et al. recently showed that miR-21 can be used in treating hyperlipidemia, as exercise also affects the expression of miR-21 and has beneficial effects on hyperlipidemia (Zhao et al., 2021). The sensitivity and specificity tests for diagnosing hypothyroidism showed a higher level of sensitivity and specificity among the miR-206 marker levels, with a sensitivity of 96.7% and a specificity of 96.7% at a cut-off value of 1.55. For subclinical disease, the sensitivity test for miR-206 and miR-181b showed the same level (100%) of sensitivity and specificity (96.7%) at a cut-off value of 1.55.

## 5 Conclusion

Finally, it was concluded that these miRNAs (miR-206, miR-181b, and miR-21) can be used as diagnostic biomarkers for hypothyroidism. TH is a significant regulator of these biomarkers, which influence related disorders such as dyslipidemia and hepatic steatosis; therefore, they may be used as therapeutic targets for controlling the previously mentioned disorders during hypothyroid disease. Further studies are required to validate these results.

## Data Availability

The original contributions presented in the study are included in the article/Supplementary material, further inquiries can be directed to the corresponding authors.
